# Association of baseline neutrophil-to-eosinophil ratio with response to nivolumab plus ipilimumab in patients with metastatic renal cell carcinoma

**DOI:** 10.1186/s40364-021-00334-4

**Published:** 2021-11-03

**Authors:** Matthew D. Tucker, Landon C. Brown, Yu-Wei Chen, Chester Kao, Nathan Hirshman, Emily N. Kinsey, Kristin K. Ancell, Kathryn E. Beckermann, Nancy B. Davis, Renee McAlister, Kerry Schaffer, Andrew J. Armstrong, Michael R. Harrison, Daniel J. George, W. Kimryn Rathmell, Brian I. Rini, Tian Zhang

**Affiliations:** 1 Vanderbilt University Medical Center, Department of Medicine, Division of Hematology and Oncology, 777 PRB, 2220 Pierce Avenue, Nashville, TN TN 37232 USA; 2 Vanderbilt-Ingram Cancer Center, VUMC, 777 PRB, 2220 Pierce Avenue, Nashville, TN NC 27710 USA; 3 Duke Cancer Institute Center for Prostate and Urologic Cancers, Departments of Medicine, Surgery, and Pharmacology and Cancer Biology, Duke University, DUMC Box 103861, Durham, NC USA; 4 Division of Medical Oncology, Department of Medicine, Duke University, DUMC 103861, Durham, NC NC 27710 USA; 5 Levine Cancer Institute, Atrium Health, Charlotte, NC USA; 6 Division of Hematology and Oncology, Department of Internal Medicine, UT Southwestern Medical Center, Dallas, TX USA

**Keywords:** Kidney neoplasms, Immunotherapy, Tumor biomarkers

## Abstract

**Background:**

The identification of biomarkers to select patients with metastatic renal cell carcinoma (mRCC) most likely to respond to combination immunotherapy (IO) is needed. We sought to investigate an association of the baseline neutrophil-to-eosinophil ratio (NER) with outcomes to nivolumab plus ipilimumab for patients with mRCC.

**Methods:**

We performed a retrospective review of patients with clear cell mRCC treated with nivolumab plus ipilimumab from Vanderbilt-Ingram Cancer Center and Duke Cancer Institute. Patients with prior receipt of immunotherapy and those without available baseline complete blood count with differential were excluded. Patients were divided into groups by the median baseline NER and analyzed for overall survival (OS), progression free survival (PFS), and objective response rate (ORR). Patients were also divided by median baseline neutrophil-to-lymphocyte ratio (NLR) and analyzed for clinical outcome. Further analyses of patients above/below the median NER and NLR were performed in subgroups of IMDC intermediate/poor risk, IMDC favorable risk, and treatment naïve patients.

**Results:**

A total of 110 patients were included: median age was 61 years and 75% were treatment naïve. The median NER (mNER) at baseline was 26.4. The ORR was 40% for patients with <mNER compared to 21.8% among patients with >mNER (OR 2.39, *p* = 0.04). The median PFS for patients with <mNER was significantly longer at 8.6 months (mo) compared to 3.2 mo for patients with >mNER (HR 0.50, *p* < 0.01). Median OS was not reached (NR) for patients with <mNER compared with 27.3 mo for patients with >mNER (HR 0.31, p < 0.01). The median NLR (mNLR) was 3.42. While patients with <mNLR showed improvement in OS (HR 0.42, *p* = 0.02), PFS and ORR did not differ compared with patients in the >mNLR group.

**Conclusions:**

A lower baseline NER was associated with improved clinical outcomes (PFS, OS, and ORR) in patients with mRCC treated with nivolumab plus ipilimumab, and prospective validation of the baseline NER as a predictive biomarker for response to immunotherapy-based combinations in mRCC is warranted.

**Supplementary Information:**

The online version contains supplementary material available at 10.1186/s40364-021-00334-4.

## Background

Immunotherapy-based combinations are now standard of care first-line systemic treatment options for patients with advanced renal cell carcinoma. Nivolumab (anti-programmed death-1, anti-PD-1) plus ipilimumab (anti-cytotoxic T lymphocyte-associated antigen 4, anti-CTLA-4) was the first immunotherapy combination to show an OS advantage versus sunitinib in the first-line setting [1]. Since then, three additional phase III clinical trials of PD-1 inhibitors have shown improved OS versus sunitinib when combined with anti-vascular endothelial growth factor (VEGF) tyrosine kinase inhibitors (TKIs): pembrolizumab plus axitinib, pembrolizumab plus lenvatinib, and nivolumab plus cabozantinib [2–4]. Extended 4-year follow-up data from CheckMate 214 showed an objective response rate (ORR) of 39.1% with median duration of response still not reached, highlighting the durability associated with response to this combination regimen [5]. The identification of biomarkers for response to immunotherapy may enable selection of patients most likely to benefit from immunotherapy alone versus those who require combination with TKIs. While several candidate biomarkers have been evaluated, none have yet been validated for routine clinical use [6].

A widely available and practical laboratory-based biomarker, the neutrophil-to-lymphocyte ratio (NLR) has been reported to be associated with improved outcomes to immunotherapy in mRCC [7–11] and is a currently available prognostic biomarker. However, the NLR has already demonstrated its prognostic value for mRCC prior to the advent of immune checkpoint inhibitors [12–19]. In addition, the NLR was also shown to be prognostic for improved disease-free survival and OS in the non-metastatic setting after nephrectomy [20, 21]. Therefore, while NLR has shown a continued role as a prognostic biomarker, its role as a predictive biomarker is uncertain in terms of differentiating subsets of patients with mRCC who are more likely to benefit from immunotherapy.

Prior studies have reported eosinophils as a biomarker for improved response to immunotherapy in melanoma [22, 23]. One retrospective review showed that patients with metastatic melanoma with a high relative eosinophil count had significantly improved outcomes when treated with immunotherapy but not with chemotherapy, suggesting a potential predictive use for baseline eosinophilia [23]. Additionally, a retrospective analysis of patients with metastatic RCC treated with nivolumab monotherapy showed that a higher baseline eosinophil count (> 0.1 k/uL) was associated with a lower risk of progression (HR 0.54, *p* = 0.042) [8]. Pre-clinical modeling has shown that tumor-associated eosinophils express chemokines, such as CCL5, CXCL9, and CXCL10, which promote recruitment of tumor reactive CD8+ T cells into the tumor microenvironment [24]. Eosinophils have also been shown to favor M1 macrophage skewing through the production of IFN-γ and TNF-α, thereby amplifying the Th1 anti-tumor immune response [25].

Based on these data, we performed a retrospective evaluation of a novel laboratory-based biomarker, the neutrophil-to-eosinophil ratio (NER), in patients with mRCC. We hypothesized that the baseline NER would be associated with clinical outcome of nivolumab plus ipilimumab in patients with mRCC. We also explored the NLR in the same population, to compare NER as a biomarker to an established prognostic biomarker.

## Methods

Patients with clear cell mRCC treated with the combination of nivolumab plus ipilimumab at the Vanderbilt-Ingram Cancer Center (VICC) or Duke Cancer Institute (DCI) were eligible for the current retrospective analysis. Patients with prior receipt of immunotherapy (anti-PD-1, anti-PDL-1, or anti-CTLA-4) and those without available pre-treatment complete blood count with differential were excluded. Patients included for analysis were required to begin treatment with combination nivolumab plus ipilimumab between the years of 2016 and 2020. The cutoff dates for data collection were December 1, 2020 and March 26, 2021 for patients treated at VICC and DCI, respectively.

Patient demographics, tumor characteristics and treatment information were collected via IRB-approved (VUMC 160979 and DCI Pro00101984) retrospective review of electronic medical records at both institutions. Patient demographics included age, race, and sex; treatment information included prior nephrectomy, prior systemic therapy, International Metastatic RCC Database Consortium (IMDC) risk score (favorable, intermediate, and poor risk), baseline complete blood count with differential, objective response rate (defined as complete or partial radiographic response using Response Evaluation Criteria in Solid Tumors version 1.1 [26]), progression free survival (PFS, defined by time from treatment initiation until death, radiographic or clinical progression), and overall survival (OS, defined as time from treatment initiation until death). Patients without progression or death at the study cutoff date or date of last follow up were censored for analysis.

Patients were divided into groups by the median baseline NER and were also divided by median baseline NLR for comparison. Patients were further divided by quartiles of baseline NER and NLR for subsequent analyses. The optimal NER cutpoint was calculated separately using the Contal and O’Quigley’s method, which uses the log- rank test statistic to estimate the cutpoint [27]. This additional method was performed to assess the accuracy of the use median compared to the optimal cutpoint for NER. Lastly, patients were divided into favorable risk and intermediate/poor risk per IMDC score and analyzed separately.

The NER was calculated by the absolute neutrophil count (ANC, number of cells × 10^3^/μL) divided by absolute eosinophil count (AEC, number of cells × 10^3^/μL). To allow the NER to be calculated for patients with an AEC of zero (ie. to avoid zero in the denominator), the AEC for these patients was adjusted to 0.01 × 10^3^/μL (the lowest baseline AEC recorded from patients from both laboratories). The NLR was calculated by the absolute neutrophil count (ANC, number of cells × 10^3^/μL) divided by the absolute lymphocyte count (ALC, number of cells × 10^3^/μL). No patients had an ALC of zero; therefore, no adjustments were necessary for NLR calculations in this study.

Descriptive statistics were used to present patient baseline demographics and clinical characteristics. The Student t-test and Chi-Square test were used to compare continuous variables and categorical variables, respectively. If the categorical variable had cells < 5, the Fisher exact test was used instead. Time to event outcomes were presented in Kaplan-Meier curves and compared with the log-rank test. Multivariable Cox regression model was used to investigate the association between NER or NLR with PFS and OS; variables included in the multivariable model were age, sex, race, IMDC risk, and prior systemic therapy.

Logistic regression analysis was used to estimate the odds ratio for having ORR. All statistical analyses were performed using SAS version 9.4 software (SAS Institute Inc., Cary, North Carolina) and survival curves were plotted with GraphPad Prism version 9.0 (GraphPad Software Inc., San Diego, California). A two-sided *p*-value < 0.05 was considered statistically significant.

## Results

### Baseline patient characteristics

A total of 162 patients with mRCC treated with nivolumab plus ipilimumab were identified (42 from VICC and 120 from DCI). After excluding patients with prior immunotherapy (*n* = 17), primary non-clear cell histology (*n* = 32), and patients without baseline NER available (n = 3), a total of 110 patients were included for analysis. Baseline characteristics of the overall cohort and separated by the baseline median NER are shown in Table 1; baseline characteristics separated by the baseline median NLR are shown in supplemental Table 1. The median age was 61 years, 86% of patients were white, 76% were male, 83% were intermediate/poor risk per IMDC, and 75% were treatment-naïve. There were no significant differences between the baseline age, race, sex, IMDC criteria, or prior systemic treatment between groups when divided by the median baseline NER. The median follow-up time for the overall cohort was 19.6 mo. Overall, there were 87 progression events and 35 deaths. The mPFS for the entire cohort was 5.6 mo (95% CI 3.2–8.6) and the mOS was NR (95% CI 27.3-NR).

**Table 1:** Baseline characteristics

	All Cohort	NER ≤ 26.4	NER > 26.4	***P***-value
**Age (median, IQR)**	60.5 (54–69)	62 (54–69)	58 (53–70)	0.32
Age ≤ 60.5 (%)	55 (50)	23 (42)	32 (58)	0.08
Age > 60.5 (%)	55 (50)	32 (58)	23 (42)	
**Race (%)**				
White	94 (85.5)	44 (47)	50 (53)	0.28
Non-White	12 (10.9)	8 (67)	4 (33)	
Unknown	4 (3.6)	3 (75)	1 (25)	
**Sex (%)**				
Male	84 (76)	43 (51)	41 (49)	0.65
Female	26 (24)	12 (46)	14 (54)	
**IMDC (%)**				
Favorable	18 (16)	11 (61)	7 (39)	0.33
Intermediate	73 (66)	37 (51)	36 (49)	
Poor	19 (17)	7 (37)	12 (63)	
**Prior systemic treatment (%)**				
Yes	28 (25)	11 (39)	17 (61)	0.19
No	82 (75)	44 (54)	38 (46)	

### Clinical outcomes when dividing patients by the median NER at baseline

**Fig. 1:**
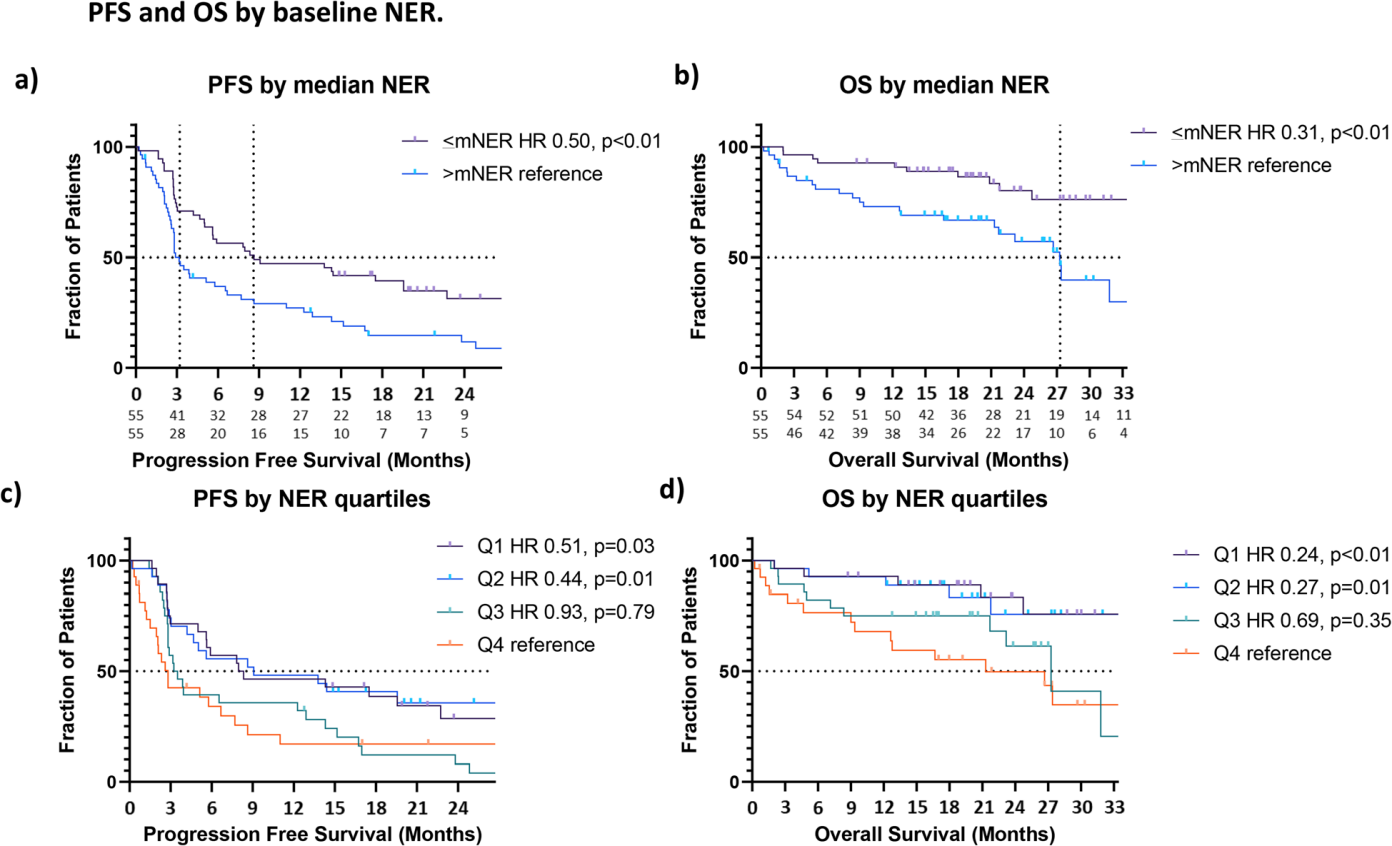
Progression free survival (PFS) and overall survival (OS) by NER. **A**) Median PFS was 8.6 mo in the <mNER group (n = 55) vs 3.2 mo in the >mNER group (n = 55) (HR 0.50, p < 0.01). **B**) Median OS was NR in the <mNER group (*n* = 55) vs 27.3 mo in the >mNER group (*n* = 55) (HR 0.31, *p* < 0.01). **C**) PFS by NER quartiles: Q1 (n = 28) HR 0.51, *p* = 0.03; Q2 (n = 27) HR 0.44, p = 0.01; Q3 (*n* = 28) HR 0.93, *p* = 0.79); Q4 (*n* = 27) reference. **D**) OS by NER quartiles: Q1 (*n* = 28) HR 0.24, *p* < 0.01; Q2 (*n* = 27) HR 0.27, p = 0.01; Q3 (n = 28) HR 0.69, *p* = 0.35; Q4 (n = 27) reference. Q1 (1st quartile), Q2 (2nd quartile), Q3 (3rd quartile), Q4 (4th quartile)

**Fig. 2:**
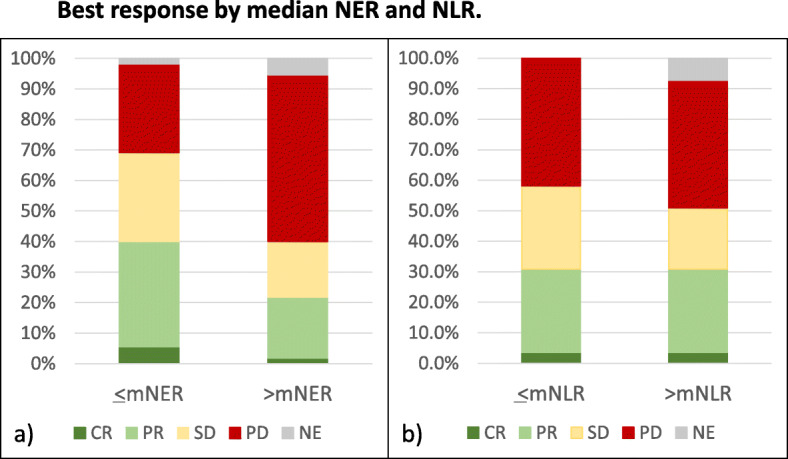
Best response by median NER and NLR. **A**) < mNER: CR = 5%, PR = 35%, SD = 29%, NE = 2%. >mNER: CR = 2%, PR = 20%, SD = 18%, PD = 55%, NE = 5%. Odds ratio for ORR = 2.39 (95% CI 1.03–5.52), *p* = 0.04. **B**) < mNLR: CR = 4%, PR = 27%, SD = 27%, PD = 42%, NE = 0%. >mNLR: CR = 4%, PR = 27%, SD = 20%, PD = 42%, NE = 7%. CR = complete response, PR = partial response, SD = stable disease, NE = not evaluable. Odds ratio for ORR = 1.00 (0.44–2.25), p = 1.00)

The median NER (mNER) at baseline was 26.4. The Contal and O’Quigley’s methods identified that the most significant cutpoints of NER for PFS fell between 25.8–27.8 (Q-statistic *p*-value: 0.0027). Patients with <mNER had longer PFS (8.6 mo vs 3.2 mo, HR 0.50, *p* < 0.01) compared with patients >mNER (Fig. 1a, Supplemental Table 2a). The median OS for patients with <mNER was not reached (NR), compared with 27.3 mo for patients with >mNER (HR 0.31, p < 0.01) (Fig. 1b, Supplemental Table 2a). Patients with <mNER had improved ORR (40.0% vs 21.8%, OR 2.39, *p* = 0.04) (Fig. 2a, Supplemental Table 3a). For illustration purposes, baseline NER was also presented as quartiles, showing favorable improvement in PFS and OS among patients with lower NER (Fig. 1c-d, Supplemental Table 4a). In multivariable Cox regression analysis after adjusting for baseline characteristics, patients with <mNER were associated with improved PFS (AHR 0.62, *p*-value: 0.04) and OS (AHR 0.32, p-value < 0.01) (Table 2).

**Table 2:** Multivariable Analysis for PFS and OS including NER

	PFS		OS	
	AHR (95% CI)	***P***-value	AHR (95% CI)	***P***-value
**NER**				
<Median	0.62 (0.39–0.98)	0.04	0.32 (0.14–0.72)	0.006
>Median	Ref		Ref	
**Age**				
Age > 60	0.82 (0.51–1.31)	0.41	1.06 (0.51–2.19)	0.88
Age < 60	Ref		Ref	
**Race**				
Non-White	0.65 (0.30–1.41)	0.28	0.42 (0.10–1.83)	0.25
White	Ref		Ref	
**Sex**				
Male	1.02 (0.61–1.69)	0.95	1.32 (0.57–3.06)	0.52
Female	Ref		Ref	
**IMDC**				
Poor	2.36 (1.08–5.14)	0.03	3.81 (0.95–15.33)	0.06
Intermediate	1.31 (0.69–2.46)	0.41	1.95 (0.57–6.71)	0.29
Favorable	Ref		Ref	
**Prior systemic therapy**				
Yes	1.78 (1.09–2.90)	0.02	0.69 (0.29–1.62)	0.39
No	Ref		Ref	

### Clinical outcomes when dividing patients by the median NLR at baseline

**Fig. 3:**
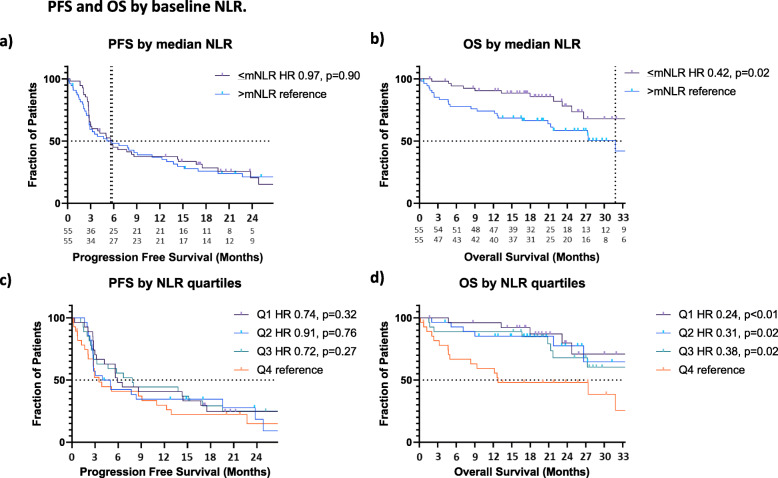
Progression free survival (PFS) and overall survival (OS) by NLR. **A**) Median PFS was 5.6 mo in the <mNLR group (*n* = 55) vs 5.8 mo in the >mNLR group (n = 55) (HR 0.97, p = 0.90). **B**) Median OS was NR in the <mNLR group (n = 55) vs 31.8 mo in the >mNLR group (n = 55) (HR 0.42, p = 0.02). **C**) PFS by NLR quartiles: Q1 (*n* = 27) HR 0.74, *p* = 0.32; Q2 (*n* = 28) HR 0.91, *p* = 0.76; Q3 (n = 27) HR 0.72, *p* = 0.27); Q4 (n = 28) reference. **D**) OS by NLR quartiles: Q1 (n = 27) HR 0.24, p < 0.01; Q2 (n = 28) HR 0.31, *p* = 0.02; Q3 (n = 27) HR 0.38, p = 0.02; Q4 (n = 28) reference. Q1 (1st quartile), Q2 (2nd quartile), Q3 (3rd quartile), Q4 (4th quartile)

The median NLR (mNLR) at baseline was 3.42. There was no statistical difference in median PFS by baseline NLR (<mNLR: 5.6 mo vs 5.8 mo in patients with >mNLR; HR 0.97, *p* = 0.90) (Fig. 3a, Supplemental Table 2b). Patients with <mNLR had longer OS (NR vs 31.8 mo; HR: 0.42, *p* = 0.02) (Fig. 3b, Supplemental Table 2b). There was no difference in ORR (30.9% vs 30.9%, OR 1.00, *p* = 1.00) (Fig. 2b, Supplemental Table 3b). When splitting NLR into quartiles, patients in the highest baseline NLR quartile (Q4) had the shortest OS, but there were no significant difference in PFS among quartiles (Fig. 3c-d, Supplemental Table 4b). In multivariable Cox regression analysis, mNLR was not associated with PFS (AHR 1.05, p-value: 0.83) or OS (AHR 0.50, *p* = 0.07) (Table 3).

**Table 3:** Multivariable Analysis for PFS and OS including NLR

	PFS		OS	
	AHR (95% CI)	***P***-value	AHR (95% CI)	***P***-value
**NLR**				
<Median	1.05 (0.68–1.63)	0.83	0.50 (0.24–1.05)	0.07
>Median	Ref		Ref	
**Age**				
Age > 60	0.75 (0.47–1.20)	0.23	0.87 (0.43–1.75)	0.69
Age < 60	Ref		Ref	
**Race**				
Non-White	0.55 (0.26–1.20)	0.13	0.34 (0.08–1.47)	0.15
White	Ref		Ref	
**Sex**				
Male	0.95 (0.57–1.57)	0.84	1.11 (0.50–2.45)	0.79
Female	Ref		Ref	
**IMDC**				
Poor	2.78 (1.29–5.99)	0.009	4.60 (1.15–18.32)	0.03
Intermediate	1.42 (0.76–2.67)	0.27	2.21 (0.66–7.44)	0.20
Favorable	Ref		Ref	
**Prior systemic therapy**				
Yes	1.93 (1.19–3.12)	0.008	0.92 (0.40–2.11)	0.84
No	Ref		Ref	

### Clinical outcomes by NER and NLR when dividing patients by IMDC risk groups

There were 92 patients with intermediate/poor risk disease per IMDC criteria. The median NER among intermediate/poor risk patients was 28.9 and the median NLR was 3.5. Patients with IMDC intermediate/poor risk disease with <mNER had significantly improved PFS (8.2 mo vs 2.9 mo, HR 0.55, *p* = 0.01) compared with patients >mNER (Supplemental Fig. 1a, Supplemental Table 5a). The median OS for patients with IMDC intermediate/poor risk disease was NR in the <mNER group vs 26.7 mo in the >mNER group (HR 0.32, *p* < 0.01) (Supplemental Fig. 1b). The ORR was numerically higher among patients in the <mNER group vs the >mNER group (37% vs 22%, OR 2.11, *p* = 0.11). In contrast, for the NLR analysis, among patients with intermediate/poor risk disease there was no significant difference in PFS between the <mNLR and > mNLR groups (4.2 mo vs 5.8 mo, HR 1.05, *p* = 0.83) (Supplemental Fig. 1c, Supplemental Table 5b). The median OS for this cohort of patients with intermediate/poor risk disease was NR in the <mNLR group and 27.4 mo in the >mNLR group (HR 0.49, *p* = 0.06) (Supplemental Fig. 1d). The ORR among patients with intermediate/poor risk disease in the <mNLR group was 28% vs 30% in the >mNLR group (OR 0.90, *p* = 0.82).

There were 18 patients with favorable risk disease per IMDC criteria. The median NER among favorable risk patients was 20.2 and the median NLR was 1.7. The mPFS among patients in the <mNER group was 34.0 mo vs 3.5 mo in the >mNER group, (HR 0.33, *p* = 0.07) (Supplemental Fig. 2a, Supplemental Table 6a). The median OS for patients with favorable risk disease was NR in both the <mNER and > mNER groups (HR 0.56 *p* = 0.64) (Supplemental Fig. 2b). The ORR was numerically higher in the <mNER group vs the >mNER group (56% vs 22%, OR 7.2, p = 0.11). Among patients with favorable risk disease, there was no significant difference in PFS between the <mNLR and > mNLR groups (5.9 mo vs 5.0 mo, HR 0.89, *p* = 0.84) (Supplemental Fig. 2c, Supplemental Table 6b). The median OS for patients with favorable risk disease was NR in both the <mNLR and > mNLR groups (HR 1.64, *p* = 0.69) (Supplemental Fig. 2d). The ORR among favorable risk patients in the <mNLR group was 33% vs 44% in the >mNLR group (OR 0.63, *p* = 0.63).

### Clinical outcomes by NER and NLR among treatment naïve patients

There were 28 patients (25%) who received prior VEGF/TKI and no patients who received prior IO, as these patients were excluded. When limiting the analysis to patients treated with first-line nivolumab plus ipilimumab, the median NER was 22.9 and the median NLR was 3.6. The mPFS among patients in the <mNER group was 14.3 mo vs 3.9 mo in the >mNER group, (HR 0.49, *p* < 0.01) (Supplemental Fig. 3a, Supplemental Table 7a). The median OS for treatment naïve patients was NR in the <mNER and 26.7 mo in the >mNER group (HR 0.37 *p* = 0.01) (Supplemental Fig. 3b). The ORR was numerically higher in the <mNER group vs the >mNER group (41% vs 29%, OR 1.7, *p* = 0.25). Among treatment naïve patients, there was no significant difference in PFS between the <mNLR and > mNLR groups (7.7 mo vs 7.9 mo, HR 0.97, *p* = 0.89) (Supplemental Fig. 3c, Supplemental Table 7b). There was also no significant difference in OS (NR vs NR, HR 0.66, *p* = 0.29) (Supplemental Fig. 3d). The ORR in the <mNLR group was 37% vs 34% in the >mNLR group (OR 1.11, *p* = 0.82).

## Discussion

To our knowledge, the current study represents the first evaluation of the baseline NER, a novel laboratory-based biomarker, with response to nivolumab plus ipilimumab in mRCC. Patients with <mNER had significantly improved PFS and OS both before and after adjusting for baseline characteristics, including IMDC criteria. While patients with <mNLR showed similar improvement in OS, there was no significant improvement in PFS or ORR compared with patients in the >mNLR group. When separated into favorable and intermediate/poor risk groups per IMDC criteria, similar improvement in PFS were observed in the <mNER group. While the improvement in PFS was not significant in the favorable risk group, this may be due to the limited sample size of this subgroup. When limited to treatment naïve patients, which represented 75% of the total cohort, both PFS and OS remained significant for baseline NER and favored the <NER group. However, baseline NLR was not associated with a significant difference in either PFS or OS among treatment naïve patients.

While the NLR was associated with improved OS for the overall cohort in this study, the fact that NLR has been consistently shown to be associated with improved survival with anti-VEGF TKIs, mTOR inhibition, immunotherapy, and post-nephrectomy supports the notion that NLR is a prognostic biomarker in advanced RCC regardless of treatment type. Unlike NLR, a lower NER was additionally associated with improved ORR and PFS. Given the improvement in PFS and ORR, the baseline NER was better able to predict for response to nivolumab plus ipilimumab than the NLR in this analysis. Therefore, further prospective validation of the NER as a predictive biomarker for immunotherapy-based combinations in mRCC is warranted.

Many pre-clinical models have shown that eosinophils play a vital role in the tumor microenvironment (TME) and response to immunotherapy through increased expression of CCL5, CXCL9, and CXCL10 [24, 25]. These chemokines promote recruitment of cytotoxic CD8+ T-lymphocytes into the TME. More recently, Cheng et al. reported that eosinophil-related markers are substantially increased in the TME following radiation exposure. Using mouse models, they showed that eosinophil infiltration into the TME was also markedly increased after radiation [28]. Furthermore, they showed that increased eosinophils were not only associated with recruitment of CD8+ T-lymphocytes into the TME, but also that eosinophils were necessary for the cytotoxic anti-tumor response observed. This work provides further evidence that baseline eosinophilia may lead to improved response to immunotherapy.

Our study has several limitations, including being retrospective and largely descriptive in nature without having a non-immunotherapy agent for comparison, such as an anti-VEGF TKI or the newer immunotherapy-TKI combinations. Given that both neutrophils and eosinophils may be affected by a variety of medications, infections, and autoimmune conditions, our study is further limited in its ability to adequately account for potential influences from these external sources. In addition, we did not have on-treatment data to compare for changes in NER or NLR while on treatment, as have been found to associate with immunotherapy outcomes in a prior study [29]. However, while our study lacks a direct validation cohort, a post-hoc analysis of the phase III randomized control trial, JAVELIN Renal 101, demonstrated that a lower NER was associated with improved ORR and PFS with combination avelumab (anti-PD-L1) and axitinib [30]. Notably, this JAVELIN Renal 101 study also provides the first evaluation of baseline NER for TKI monotherapy, showing no significant difference in PFS by median NER but significant improvement in OS by median NER. Therefore, like NLR, NER may have prognostic importance regardless of treatment type, but may have predictive importance for patients most likely to respond to immunotherapy-based combinations. If this association with nivolumab plus ipilimumab is prospectively validated, the NER may help select which patients are most likely to benefit from immunotherapy alone, potentially sparing the need for adverse effects of long term TKI use.

Particularly relevant are our findings of the predictive nature of NER in IMDC favorable risk disease, where nivolumab plus ipilimumab has less clear absolute benefit compared with TKI monotherapy but may spare a patient long-term TKI use. This distinguishing feature to select patients with clinically meaningful and durable responses to first-line nivolumab plus ipilimumab may define a subset of patients who do not necessarily need upfront TKI use. Measuring the NER is clinically facile and with further prospective testing, the NER biomarker has potential to select patients who are most likely to respond to combination nivolumab plus ipilimumab. Future prospective studies exploring the predictive utility of the baseline NER should also measure serum levels of CCL5, CXCL9, and CXCL10 at baseline and on treatment. These measurements may help determine the underlying mechanism of why patients with lower baseline NER appear to have improved outcomes and also help identify future targets and mechanisms to increase the chances of patients with high baseline NER to respond to immunotherapy.

## Conclusion

Using a multi-institutional cohort of mRCC patients treated with nivolumab plus ipilimumab, we showed that NER was significantly associated with improved PFS, OS, and ORR, whereas NLR was only associated with improved OS. Therefore, we suggest that prospective validation of the NER as a predictive biomarker for response to immunotherapy-based combinations in mRCC is warranted, along with further investigation of eosinophils in the tumor microenvironment.

## Supplementary Information

**Additional file 1:.**

**Additional file 2: Supplemental Figure 1:** PFS and OS by median NER and NLR among intermediate/poor risk. A) Median PFS was 8.2 mo in the <mNER group (*n*=46) vs 2.9 mo in the >mNER group (n=46) (HR 0.55, *p*=0.01). B) Median OS was NR in the <mNER group (n=46) vs 26.7 mo in the >mNER group (n=46) (HR 0.32, *p*<0.01). C) Median PFS was 4.2 mo in the <mNLR group (n=46) vs 5.8 mo in the >mNLR group (n=46) (HR 1.05, *p*=0.83). D) Median OS was NR in the <mNLR group (n=46) vs 27.4 mo in the >mNLR group (n=46) (HR 0.49, *p*=0.06).

**Additional file 3: Supplemental Figure 2:** PFS and OS by median NER and NLR among favorable risk. A) Median PFS was 34.0 mo in the <mNER group (*n*=9) vs 3.5 mo in the >mNER group (n=9) (HR 0.33, *p*=0.07). B) Median OS was NR in the <mNER group (n=9) vs NR in the >mNER group (n=9) (HR 0.56, *p*=0.64). C) Median PFS was 5.9 mo in the <mNLR group (n=9) vs 5.0 mo in the >mNLR group (n=9) (HR 0.89, *p*=0.84). D) Median OS was NR in the <mNLR group (n=9) vs NR in the >mNLR group (n=9) (HR 1.64, *p*=0.69).

**Additional file 4: Supplemental Figure 3:** PFS and OS by median NER and NLR among treatment naïve patients: A) Median PFS was 14.3 mo in the <mNER group (*n*=41) vs 3.9 mo in the >mNER group (n=41) (HR 0.49, p<0.01). B) Median OS was NR in the <mNER group (n=41) vs 26.7 mo in the >mNER group (n=41) (HR 0.37, p=0.01). C) Median PFS was 7.7 mo in the <mNLR group (n = 41) vs 7.9 mo in the >mNLR group (n = 41) (HR 0.97, *p* = 0.89). D) Median OS was NR in the <mNLR group (n = 41) vs NR in the >mNLR group (n = 41) (HR 0.66, *p* = 0.29).

## Data Availability

The datasets used and analyzed during the current study are available from the corresponding author on reasonable request.

## Abbreviations

AEC: Absolute eosinophil count
ALC: Absolute lymphocyte count
ANC: Absolute neutrophil count
CTLA-4: Cytotoxic T lymphocyte-associated antigen 4
DCI: Duke Cancer Institute
IO: Immunotherapy
IMDC: International Metastatic RCC Database Consortium
mRCC: Metastatic renal cell carcinoma
NER: Neutrophil-to-eosinophil ratio
NLR: Neutrophil-to-lymphocyte ratio
NR: Not reached
ORR: Objective response rate
OS: Overall survival
PD-1: Programmed death-1
PFS: Progression free survival
Q: Quartile
TME: Tumor microenvironment
TKIs: Tyrosine kinase inhibitors
VICC: Vanderbilt-Ingram Cancer Center
VEGF: Vascular endothelial growth factor
