# Supplementary material for: Association of baseline neutrophil-to-eosinophil ratio with response to nivolumab plus ipilimumab in patients with metastatic renal cell carcinoma
Source: Biomark Res. 2021 Nov 3;9:80. doi: 10.1186/s40364-021-00334-4 (PMC8564988; doi:10.1186/s40364-021-00334-4)
Supplement: Supplementary file 1 — Additional file 1:. [file 40364_2021_334_MOESM1_ESM.docx]

**Supplementary Table 1: Baseline characteristics by NLR**

|  | **All Cohort** | **NLR≤3.42** | **NLR>3.42** | **P-value** |
| --- | --- | --- | --- | --- |
| **Age (median, IQR)** | 60.5 (54-69) | 61 (53-68) | 59 (54-69) | 0.97 |
| Age ≤ 60.5 (%) | 55 (50) | 25 (46) | 30 (54) | 0.34 |
| Age > 60.5 (%) | 55 (50) | 30 (55) | 25 (45) |  |
|  |  | | | |
| **Race (%)** |  |  | |  |
| White | 94 (85.5) | 45 (48) | 49 (52) | 0.43 |
| Non-White | 12 (10.9) | 8 (67) | 4 (33) |  |
| Unknown | 4 (3.6) | 2 (50) | 2 (50) |  |
|  |  | | | |
| **Sex (%)** |  |  | |  |
| Male | 84 (76) | 42 (76) | 42 (76) | 1.00 |
| Female | 26 (24) | 13 (24) | 13 (24) |  |
|  |  | | | |
| **IMDC (%)** |  |  |  |  |
| Favorable | 18 (16) | 11 (61) | 7 (39) | 0.02 |
| Intermediate | 73 (66) | 40 (55) | 33 (45) |  |
| Poor | 19 (17) | 4 (21) | 15 (79) |  |
|  |  | | | |
| **Prior systemic treatment (%)** |  |  |  |  |
| Yes | 28 (25) | 38 (46) | 44 (54) | 0.19 |
| No | 82 (75) | 17 (61) | 11 (39) |  |

**Supplementary Table 2: Univariate HR for PFS and OS by median NER and NLR.**

1. **NER**

|  | **PFS** | | **OS** | |
| --- | --- | --- | --- | --- |
|  | **HR (95% CI)** | **P-value** | **HR (95% CI)** | **P-value** |
| NER< median | 0.50 (0.32-0.77) | 0.002 | 0.31 (0.15-0.64) | 0.002 |
| NER> median | Ref |  | Ref |  |

1. **NLR**

|  | **PFS** | | **OS** | |
| --- | --- | --- | --- | --- |
|  | **HR (95% CI)** | **P-value** | **HR (95% CI)** | **P-value** |
| NLR< median | 0.97 (0.64-1.49) | 0.90 | 0.42 (0.21-0.87) | 0.02 |
| NLR> median | Ref |  | Ref |  |

**Supplementary Table 3: Univariate Odds ratio for ORR**

1. **NER**

|  | **OR (95% CI)** | **P-value** |
| --- | --- | --- |
| NER< median | 2.39 (1.03-5.52) | 0.04 |
| NER> median | Ref |  |

1. **NLR**

|  | **OR (95% CI)** | **P-value** |
| --- | --- | --- |
| NLR< median | 1.00 (0.44-2.25) | 1.00 |
| NLR> median | Ref |  |

**Supplementary Table 4: Univariable Results for PFS and OS by NER and NLR quartiles.**

1. **NER**

|  | **PFS (HR, 95% CI)** | **P-value** | **OS (HR, 95% CI)** | **P-value** |
| --- | --- | --- | --- | --- |
| Q1 | 0.51 (0.28-0.93) | 0.03 | 0.24 (0.09-0.67) | 0.006 |
| Q2 | 0.44 (0.23-0.84) | 0.01 | 0.27 (0.10-0.75) | 0.01 |
| Q3 | 0.93 (0.52-1.64) | 0.79 | 0.69 (0.31-1.51) | 0.35 |
| Q4 | Ref |  | Ref |  |

1. **NLR**

|  | **PFS (HR, 95% CI)** | **P-value** | **OS (HR, 95% CI)** | **P-value** |
| --- | --- | --- | --- | --- |
| Q1 | 0.74 (0.41-1.34) | 0.32 | 0.24 (0.09-0.65) | 0.005 |
| Q2 | 0.91 (0.51-1.64) | 0.76 | 0.31 (0.12-0.80) | 0.015 |
| Q3 | 0.72 (0.40-1.29) | 0.27 | 0.38 (0.16-0.88) | 0.024 |
| Q4 | Ref |  | Ref |  |

**Supplementary Table 5: Univariate HR for PFS and OS by median NER and NLR (Intermediate/Poor Risk):**

1. **NER Intermediate/Poor Risk**

|  | **PFS** | | | **OS** | | | **ORR** | |
| --- | --- | --- | --- | --- | --- | --- | --- | --- |
|  | **HR (95% CI)** | **P-value** | **Median Survival (months)** | **HR (95% CI)** | **P-value** | **Median Survival (months)** | **OR (95% CI)** | **P-value** |
| <Median | 0.55 (0.34-0.87) | 0.011 | 8.15 | 0.32 (0.15-0.68) | 0.003 | NR | 2.11 (0.84-5.30) | 0.11 |
| ≥Median | Ref |  | 2.9 | Ref |  | 26.7 |  |  |

1. **NLR Intermediate/Poor Risk**

|  | **PFS** | | | **OS** | | | **ORR** | |
| --- | --- | --- | --- | --- | --- | --- | --- | --- |
|  | **HR (95% CI)** | **P-value** | **Median Survival (months)** | **HR (95% CI)** | **P-value** | **Median Survival (months)** | **OR (95% CI)** | **P-value** |
| <Median | 1.05 (0.66-1.67) | 0.83 | 4.20 | 0.49 (0.24-1.02) | 0.06 | NR | 0.90 (0.37-2.21) | 0.82 |
| ≥Median | Ref |  | 5.76 | Ref |  | 27.4 |  |  |

**Supplementary Table 6: Univariate HR for PFS and OS by median NER and NLR (Favorable):**

1. **NER Favorable Risk:**

|  | **PFS** | | | **OS** | | | **ORR** | |
| --- | --- | --- | --- | --- | --- | --- | --- | --- |
|  | **HR (95% CI)** | **P-value** | **Median Survival (months)** | **HR (95% CI)** | **P-value** | **Median Survival (months)** | **OR (95% CI)** | **P-value** |
| <Median | 0.33 (0.10-1.11) | 0.074 | 34 | 0.56 (0.05-6.32) | 0.64 | NR | 7.20 (0.64-81.50) | 0.11 |
| ≥Median | Ref |  | 3.5 | Ref |  | NR | Ref |  |

1. **NLR Favorable Risk:**

|  | **PFS** | | | **OS** | |  | **ORR** | |
| --- | --- | --- | --- | --- | --- | --- | --- | --- |
|  | **HR (95% CI)** | **P-value** | **Median Survival (months)** | **HR (95% CI)** | **P-value** | **Median Survival (months)** | **OR (95% CI)** | **P-value** |
| <Median | 0.89 (0.28-2.78) | 0.84 | 5.9 | 1.64 (0.15-18.25) | 0.69 | NR | 0.63 (0.09-4.22) | 0.63 |
| ≥Median | Ref |  | 5.03 | Ref |  | NR |  |  |

**Supplementary Table 7: Univariate HR for PFS and OS by median NER and NLR among treatment naïve patients.**

1. **NER among treatment naïve patients**

|  | **PFS** | | | **OS** | | | **ORR** | |
| --- | --- | --- | --- | --- | --- | --- | --- | --- |
|  | **HR (95% CI)** | **P-value** | **Median Survival (months)** | **HR (95% CI)** | **P-value** | **Median Survival (months)** | **OR (95% CI)** | **P-value** |
| <Median | 0.49 (0.29-0.83) | <0.01 | 14.3 | 0.37 (0.16-0.82) | 0.01 | NR | 1.71 (0.69-4.28) | 0.25 |
| ≥Median | Ref |  | 3.9 | Ref |  | 26.7 |  |  |

1. **NLR among treatment naïve patients**

|  | **PFS** | | | **OS** | | | **ORR** | |
| --- | --- | --- | --- | --- | --- | --- | --- | --- |
|  | **HR (95% CI)** | **P-value** | **Median Survival (months)** | **HR (95% CI)** | **P-value** | **Median Survival (months)** | **OR (95% CI)** | **P-value** |
| <Median | 0.97 (0.58-1.61) | 0.89 | 7.7 | 0.66 (0.31-1.43) | 0.29 | NR | 1.11 (0.45-2.75) | 0.82 |
| ≥Median | Ref |  | 7.9 | Ref |  | NR |  |  |
