# Supplementary figures and images for: Association of baseline neutrophil-to-eosinophil ratio with response to nivolumab plus ipilimumab in patients with metastatic renal cell carcinoma
Source: Biomark Res. 2021 Nov 3;9:80. doi: 10.1186/s40364-021-00334-4 (PMC8564988; doi:10.1186/s40364-021-00334-4)

Supplemental Figure 1: PFS and OS by median NER and NLR among intermediate/poor risk.

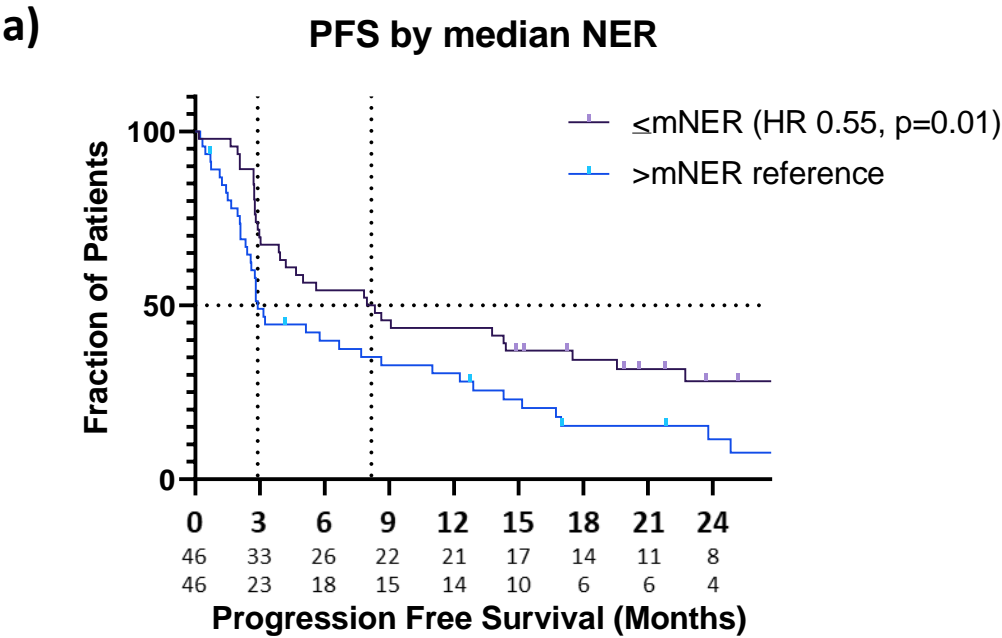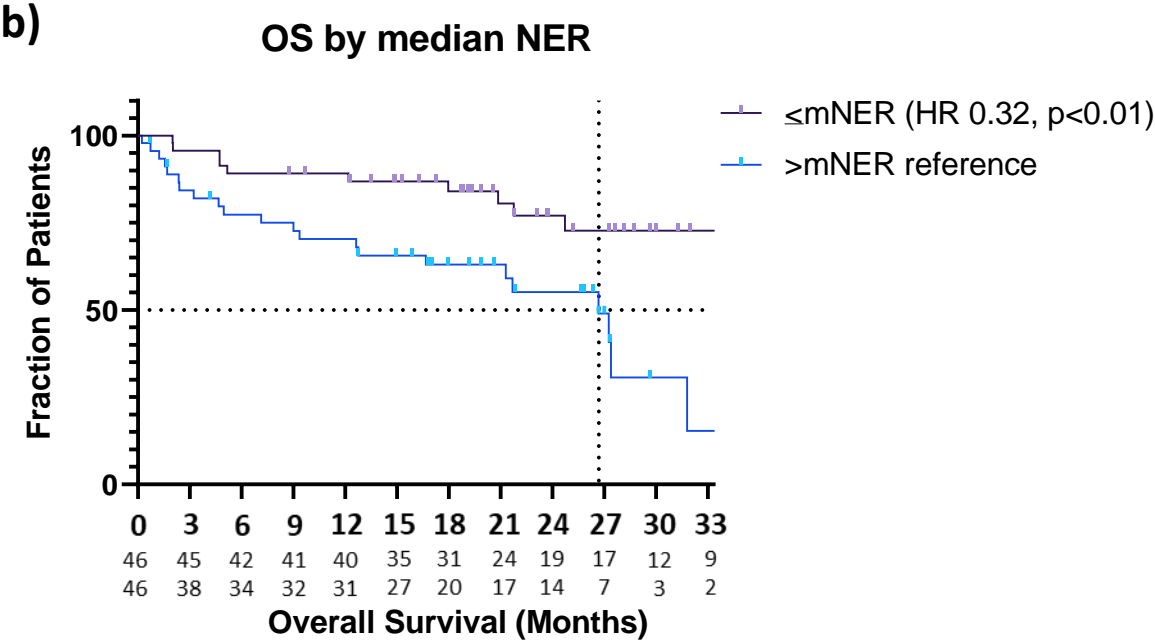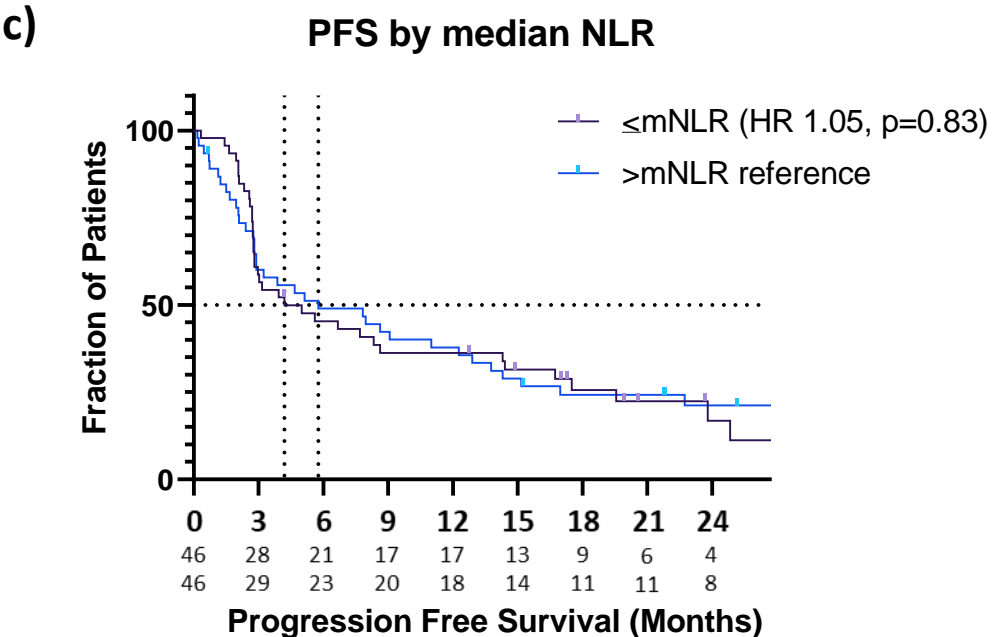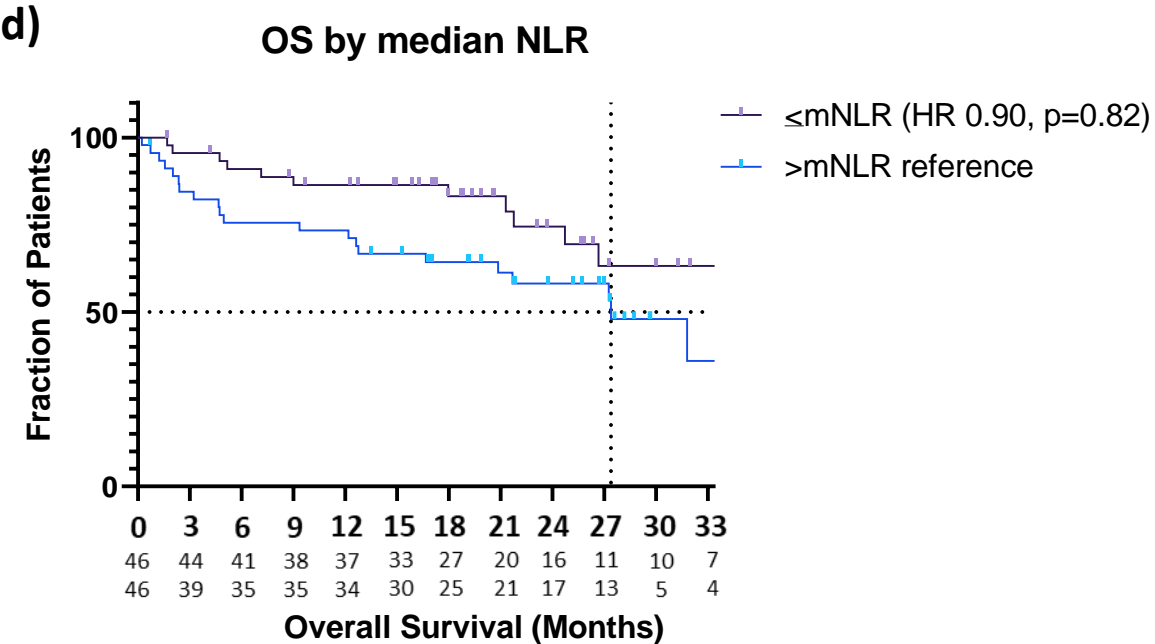

Supplement: Supplementary file 2 — Additional file 2: Supplemental Figure 1: PFS and OS by median NER and NLR among intermediate/poor risk. A) Median PFS was 8.2 mo in the mNER group (n=46) (HR 0.55, p=0.01). B) Median OS was NR in the mNER group (n=46) (HR 0.32, p<0.01). C) Median PFS was 4.2 mo in the mNLR group (n=46) (HR 1.05, p=0.83). D) Median OS was NR in the mNLR group (n=46) (HR 0.49, p=0.06). [file 40364_2021_334_MOESM2_ESM.pdf]

Supplemental Figure 2: PFS and OS by median NER and NLR among favorable risk.

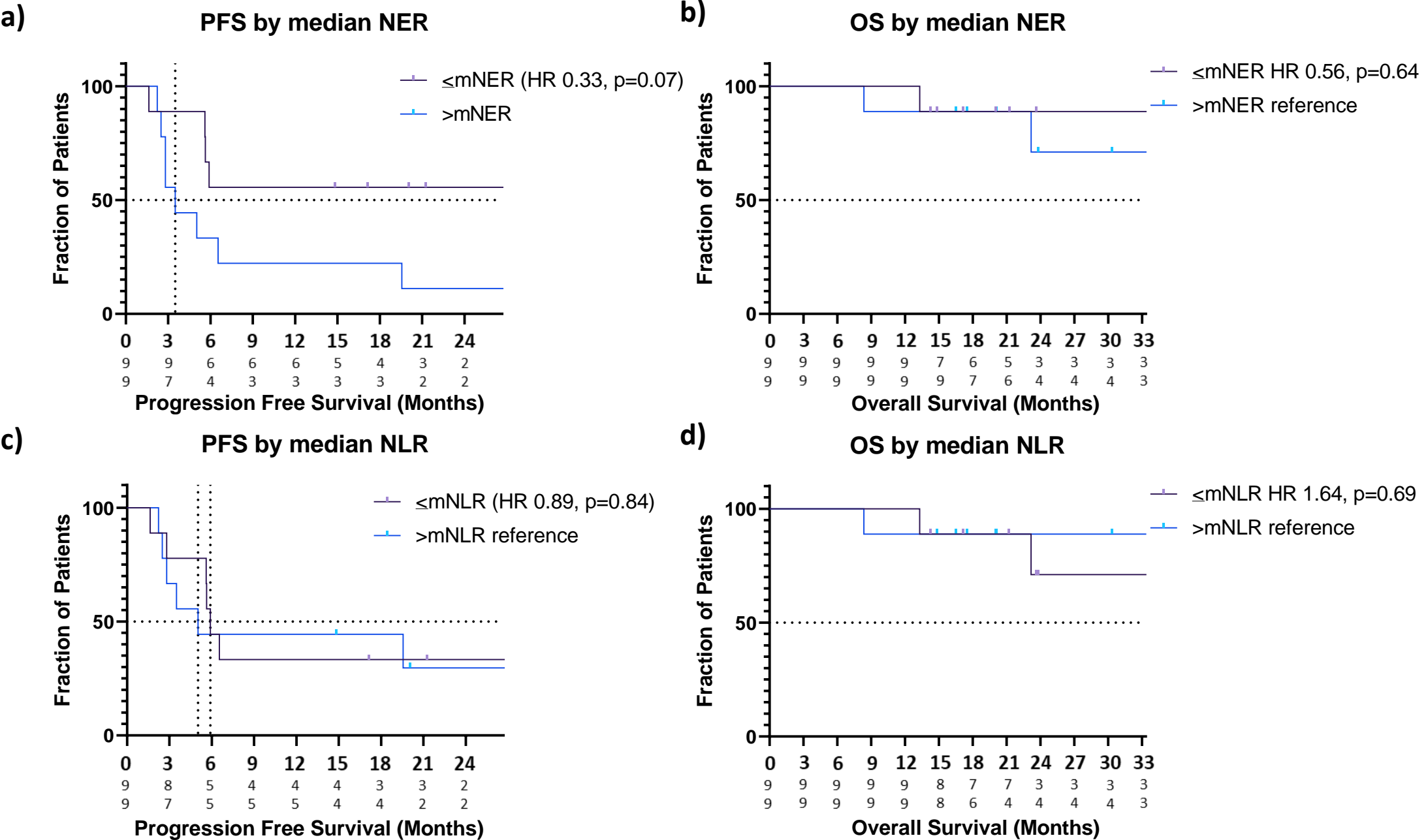

Supplement: Supplementary file 3 — Additional file 3: Supplemental Figure 2: PFS and OS by median NER and NLR among favorable risk. A) Median PFS was 34.0 mo in the mNER group (n=9) (HR 0.33, p=0.07). B) Median OS was NR in the mNER group (n=9) (HR 0.56, p=0.64). C) Median PFS was 5.9 mo in the mNLR group (n=9) (HR 0.89, p=0.84). D) Median OS was NR in the mNLR group (n=9) (HR 1.64, p=0.69). [file 40364_2021_334_MOESM3_ESM.pdf]

Supplemental Figure 3: PFS and OS by median NER and NLR among treatment naïve patients.

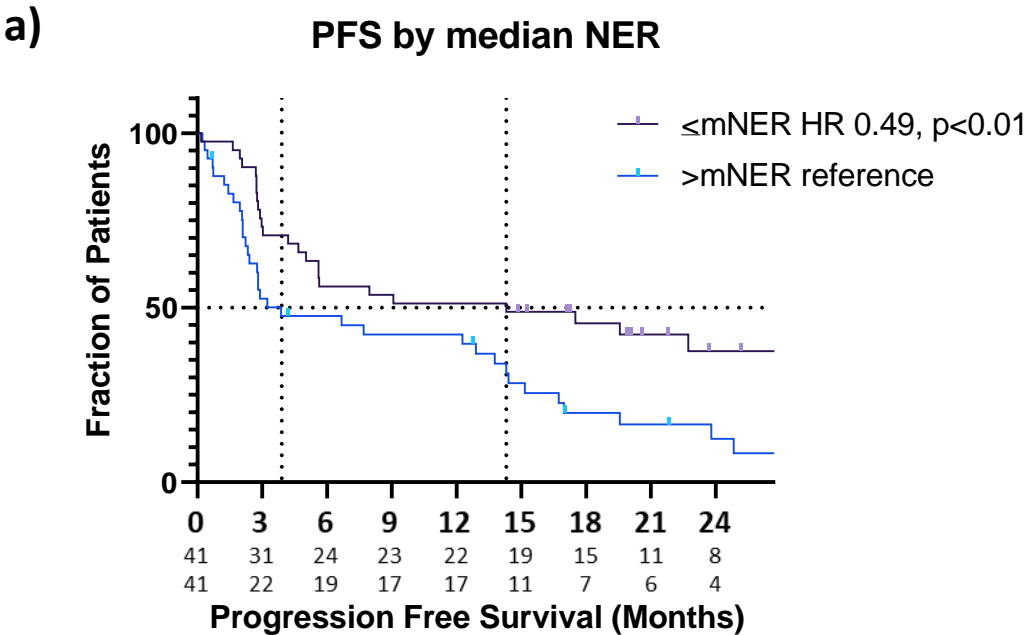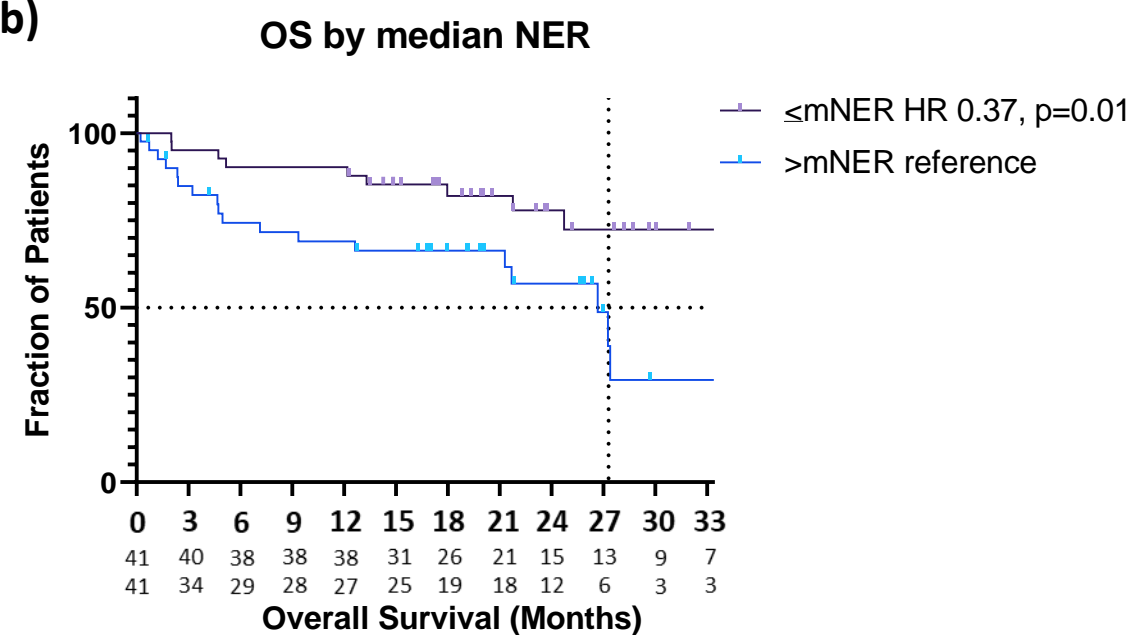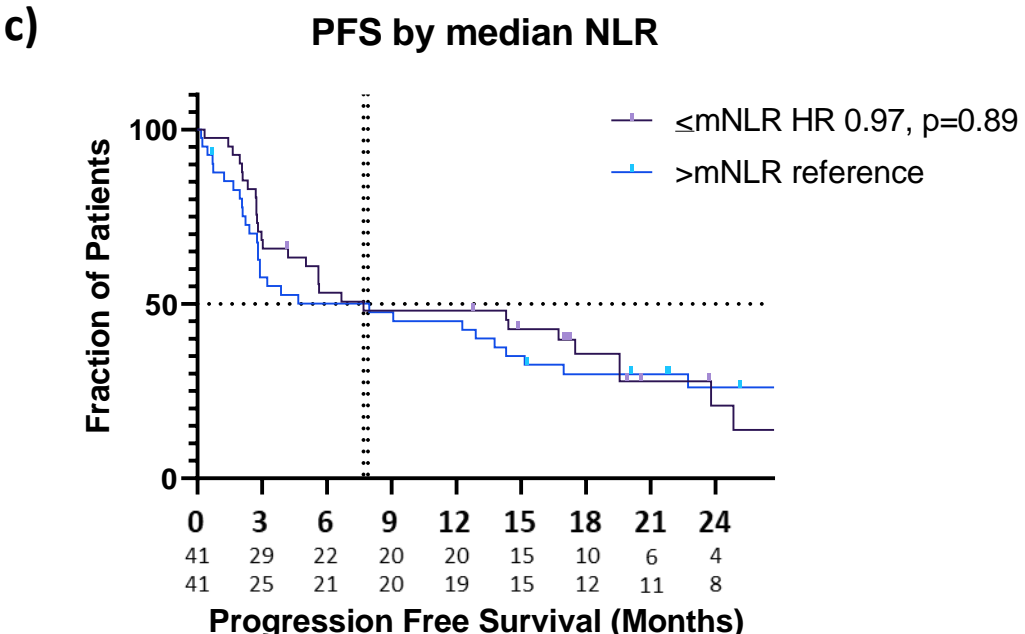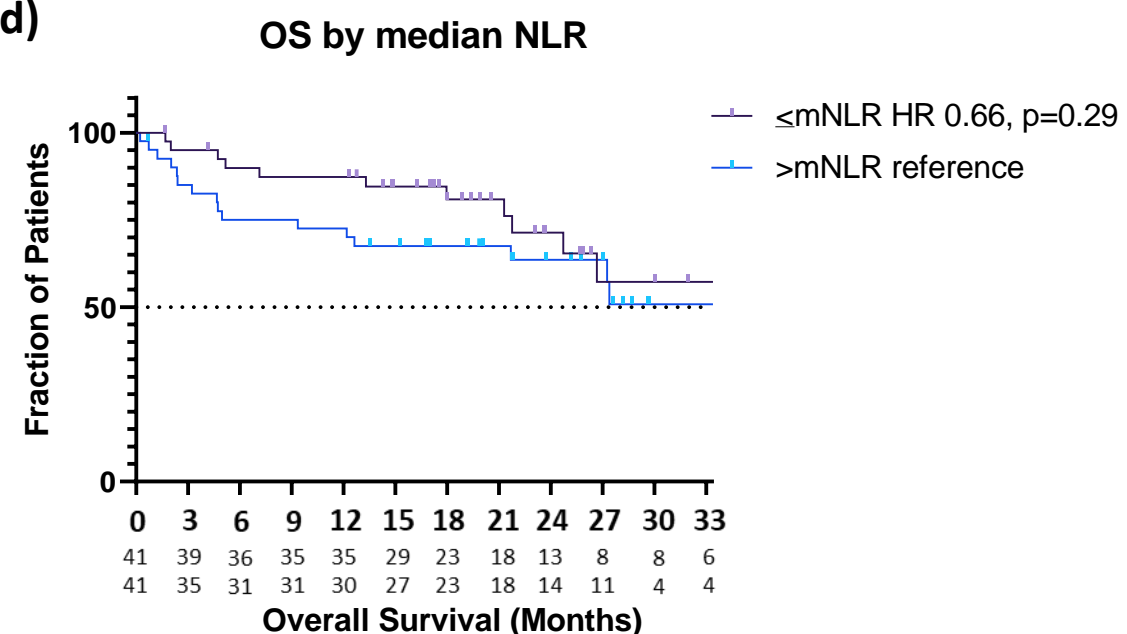

Supplement: Supplementary file 4 — Additional file 4: Supplemental Figure 3: PFS and OS by median NER and NLR among treatment naïve patients: A) Median PFS was 14.3 mo in the mNER group (n=41) (HR 0.49, p<0.01). B) Median OS was NR in the mNER group (n=41) (HR 0.37, p=0.01). C) Median PFS was 7.7 mo in the mNLR group (n = 41) (HR 0.97, p = 0.89). D) Median OS was NR in the mNLR group (n = 41) (HR 0.66, p = 0.29). [file 40364_2021_334_MOESM4_ESM.pdf]
